# Different Doses of Ilaprazole for Dual Therapy Versus Bismuth‐Quadruple Therapy for *Helicobacter pylori* Infection: A Three‐Arm, Randomized Clinical Trial

**DOI:** 10.1111/hel.70086

**Published:** 2025-11-10

**Authors:** Qiang Han, Ming Zu, Shiyu Du, Yinyan Guo, Yuanmin Zhu, Yali Wang, Xiuqing Liu, Hongwei Guo, Yanli Cheng, Shigang Ding

**Affiliations:** ^1^ Department of Gastroenterology The First Hospital of Tsinghua University Beijing China; ^2^ Department of Gastroenterology Peking University Third Hospital Beijing China; ^3^ Department of Gastroenterology China‐Japan Friendship Hospital Beijing China; ^4^ Department of Gastroenterology Beijing Haidian Hospital Beijing China; ^5^ Department of Gastroenterology Aerospace Center Hospital Beijing China

**Keywords:** bismuth‐quadruple therapy, clinical trial, dual therapy, *H. pylori*, ilaprazole

## Abstract

**Background:**

Current *Helicobacter pylori (H. pylori)* eradication regimens—standard triple, bismuth‐containing quadruple, and non‐bismuth quadruple therapies—still face issues such as adverse effects and poor compliance. High‐dose dual therapy, with its simplicity, lower pill burden, and comparable efficacy, has recently gained attention. However, existing studies are largely single‐center with small sample sizes and lack sufficient evidence‐based support. This study aims to further evaluate the efficacy and safety of ilaprazole‐based dual and quadruple therapies for *H. pylori* eradication.

**Methods:**

This was a prospective, multicenter, randomized controlled, non‐inferiority clinical trial conducted at five hospitals from January to November 2023. A total of 480 patients were randomly assigned to one of three treatment groups for 14 days: Group A (ilaprazole 5 mg, amoxicillin 1 g, clarithromycin 500 mg, and bismuth potassium citrate 220 mg, all administered twice daily), Group B (ilaprazole 5 mg twice daily and amoxicillin 1 g three times daily), and Group C (ilaprazole 10 mg twice daily and amoxicillin 1 g three times daily). The eradication rates, adverse events, and patient compliance were recorded.

**Results:**

The eradication rates of *H. pylori* in Groups A, B, and C were 76.9%, 88.1%, and 88.7% (*p* = 0.004) by intention‐to‐treat analysis, respectively, and 78.3%, 90.4%, and 91.0% (*p* = 0.001) using modified intention‐to‐treat analysis, and 81.8%, 92.1%, 92.2% (*p* = 0.005) by per‐protocol analysis. The adverse event rates were 28.0%, 10.2%, 10.8% in Groups A, B, and C, respectively (*p <* 0.001), while patient compliance rates were 91.1%, 98.1%, 98.7%, respectively (*p =* 0.001). Sex, smoking history, alcohol intake, and sharing tableware or cups did not affect the efficacy of the three treatment regimens.

**Conclusions:**

The standard‐ or high‐dose dual therapy with ilaprazole demonstrated superior efficacy, safety, and patient compliance compared to quadruple therapy. No significant differences were observed between these dual therapies, which are expected to become promising alternatives for the primary treatment of *H. pylori* infection.

**Trial Registration:**

Chinese Clinical Trial Registry: ChiCTR2400086862

## 1 Introduction

*Helicobacter pylori* ( *H. pylori* ) infection, along with its associated diseases such as gastritis, peptic ulcer, and gastric cancer, poses a global health challenge. The prevalence of this infection reaches up to 50% worldwide [1]. Since 2012, the Chinese consensus has established bismuth‐containing quadruple therapy as a first‐line treatment for *H. pylori* infection. The protocol incorporating amoxicillin and clarithromycin is widely favored for its relatively superior safety profile and ease of availability [2]. Recent epidemiological surveys have shown that the infection rate of *H. pylori* in China has dropped to 44.2% [3]. However, the widespread use of antibiotics has resulted in increased resistance, complicating the eradication process and resulting in treatment failures. It has become imperative to find treatment options with significant efficacy, fewer adverse effects, and improved patient compliance. The resistance of *H. pylori* to clarithromycin and metronidazole has remained high and continues to increase, while resistance to levofloxacin, amoxicillin, tetracycline and furazolidone has remained stable [4]. This indicates that incorporating amoxicillin into treatment protocols may provide a promising strategy.

High‐dose amoxicillin dual therapy, which has demonstrated high eradication rates and good adherence, has recently been adopted in China. The 2022 Chinese national clinical practice guideline on *H. pylori* eradication treatment recommends dual therapy as both the initial and second‐line eradication treatment [5]. Considering that the efficacy of eradication relies on improvement of acid inhibition along with current antibiotics [6], a double dose of proton pump inhibitor (PPI) was recommended to enhance acid suppression.

Ilaprazole, a third‐generation PPI, was launched in China in 2007. It has been widely used for treating duodenal ulcers, reflux esophagitis, and as part of *H. pylori* eradication therapy, at a standard dosage of 10 mg per day. Unlike previous generations of PPIs, the clinical efficacy of ilaprazole is not influenced by individual pharmacogenetic variations in CYP450 enzymes. It exhibits CYP2C19‐independent pharmacokinetics (PK) and pharmacodynamics (PD), effectively reducing nocturnal acid breakthrough [7]. Therefore, ilaprazole is regarded as a premier medication for the eradication of *H. pylori* , with a reported eradication rate of 93.0% when used in a 14‐day dual therapy regimen with Amoxicillin [8].

However, research on dual therapy of ilaprazole and amoxicillin is limited, and no studies have been conducted to ascertain the optimal dosage of ilaprazole for this regimen. To fill this gap, we conducted a prospective, multicenter, randomized controlled, non‐inferiority clinical trial to evaluate the efficacy, safety, and adherence of dual therapy with different doses of ilaprazole, compared to traditional quadruple therapy containing amoxicillin and clarithromycin. The aim of this study is to provide a more effective treatment regimen.

## 2 Materials & Methods

### 2.1 Study Design

This was a prospective, multicenter, randomized controlled, non‐inferiority clinical trial designed to evaluate the efficacy, safety, and compliance of ilaprazole‐based dual therapy (at different doses) compared to bismuth‐containing quadruple therapy for *H. pylori* eradication. The trial was conducted from January to November 2023 at the First Hospital of Tsinghua University, Peking University Third Hospital, China‐Japan Friendship Hospital, Beijing Haidian Hospital, and Aerospace Center Hospital.

### 2.2 Ethical Considerations

The study protocol has been approved by the Medical Ethics Committee of the First Hospital of Tsinghua University (Approval number: [2022]: No. 52) and was conducted following the principles of the Declaration of Helsinki and standards of good clinical practice. It was registered in the Chinese Clinical Trials Registration Center (www.chictr.org.cn, ChiCTR2400086862). Eligible participants provided a signed informed consent form.

### 2.3 Patients

Consecutive patients, aged 18–70, who tested positive for *H. pylori* infection via ^13^C‐urea breath test (^13^C‐UBT) or rapid urease test of gastric mucosal tissue, or positive pathological staining were eligible for enrollment. Exclusion criteria included: (1) patients who have previously received *H. pylori* eradication therapy; (2) patients who have taken drugs such as PPIs, H_2_ receptor antagonists, bismuth preparations, or antibiotics, in the 4 weeks before enrollment; (3) patients who were diagnosed with digestive tract tumors, severe liver or kidney dysfunction, cardiovascular and cerebrovascular diseases, mental illness, any drug allergy or intolerance or underwent upper gastrointestinal surgery; (4) patients who were pregnant or had other medical conditions that may increase adverse treatment reactions (e.g., alcohol abuse).

### 2.4 Randomization and Intervention

Eligible patients were randomly assigned in a 1:1:1 ratio to one of three treatment groups using a centrally generated, computer‐based randomization sequence. Randomization was performed by an independent statistician who was blinded to group allocation. The control group was ilaprazole 5 mg quadruple group. The experimental groups included two regimens, ilaprazole 5 mg dual group and ilaprazole 10 mg dual group. The three groups were as follows: Group A (Ilaprazole 5 mg quadruple group): Ilaprazole 5 mg twice daily (before breakfast/dinner), amoxicillin 1000 mg twice daily (after breakfast/dinner), clarithromycin 500 mg twice daily (after breakfast/dinner), and bismuth potassium citrate 220 mg twice daily (before breakfast/dinner) for 14 days; Group B (Ilaprazole 5 mg dual group): Ilaprazole 5 mg twice daily (before breakfast/dinner) and amoxicillin 1000 mg three times daily (after breakfast/lunch/dinner) for 14 days; and Group C (Ilaprazole 10 mg dual group): Ilaprazole 10 mg twice daily (before breakfast/dinner) and amoxicillin 1000 mg three times daily (after breakfast/lunch/dinner) for 14 days. Drug administration timing (before or after meals) was standardized and explained to patients in detail. All study drugs were obtained from certified pharmaceutical companies and passed quality consistency evaluations. After completing the treatment, patients were followed up within 1–3 days to assess medication adherence and record any adverse events. Eradication of *H. pylori* was assessed using a ^13^C‐UBT performed 4–6 weeks after the end of treatment. Baseline demographic and clinical characteristics were recorded at enrollment. For analytical purposes, cigarette smoking was defined as smoking more than one pack per week, and alcohol drinking was defined as consuming more than 50 g of alcohol per day in the past six months.

The study drug information was as follows: ilaprazole (Ilaprazole Enteric‐coated Tablets 5 mg/tablet; Livzon Pharmaceutical Group Inc.), amoxicillin (Amoxicillin Capsules 250 mg/capsule; Zhejiang Conba Pharmaceutical Co. Ltd.), clarithromycin (Clarithromycin Tablets 250 mg/tablet; Sunshine Lake Pharma Co. Ltd.) and bismuth potassium citrate (Bismuth Potassium Citrate Capsules 110 mg/capsule; Livzon Pharmaceutical Group Inc.).

### 2.5 Outcomes

The primary outcome was *H. pylori* eradication rate, evaluated by: Intention‐to‐treat (ITT): including all randomized patients; Modified intention‐to‐treat (mITT): including only those who received at least one dose of the medication and completed the ^13^C‐UBT as the endpoint assessment; and per‐protocol (PP): including the participants who were fully adherent to the study protocol and excluding cases with poor compliance. The secondary outcomes included the incidence of adverse events and medication compliance. Medication compliance (%) = (number of drug tablets dispensed – number of drug tablets remaining) / (number of drug tablets taken) × 100%, if compliance (%) < 80% then it is considered poor medication compliance and these patients were excluded from the PP analysis [9].

### 2.6 Sample Size Calculation and Statistical Analysis

The sample size of this study was calculated using PASS (version 16). This trial is a parallel, three‐arm, non‐inferiority design. Based on the reported eradication rate of bismuth quadruple therapy [10, 11], we assumed an average *H. pylori* eradication rate of 90% for Ilaprazole 5 mg Quadruple group. Using the non‐inferiority margin δ = −0.1 (−10%), α = 0.025 (one‐sided),β = 0.2, and a dropout rate of 10%, the estimated sample size for each group was at least 158 to achieve 80% power. Ultimately, it was decided to enroll 160 participants in each group, resulting in a total sample size of 480 for the trial. Measurement data conforming to a normal distribution are expressed as Means ± Standard Deviation ($\bar{x}$± s). Between‐group comparisons of measurement data were performed using analysis of variance (ANOVA). Categorical data are expressed as frequencies and percentages. Between‐group comparisons of categorical data were performed using chi‐square tests or Fisher's exact probability tests, as appropriate. Odds ratios (ORs) with corresponding 95% confidence intervals (CIs) were calculated using a binary logistic regression model to evaluate differences in eradication rates between groups. All statistical analyses were performed after database lock, with no interim analyses conducted using IBM SPSS statistical software version 21.0 (IBM Inc., New York, USA). A *P‐*value < 0.05 was considered statistically significant.

## 3 Results

### 3.1 Baseline Characteristics

**FIGURE 1:**
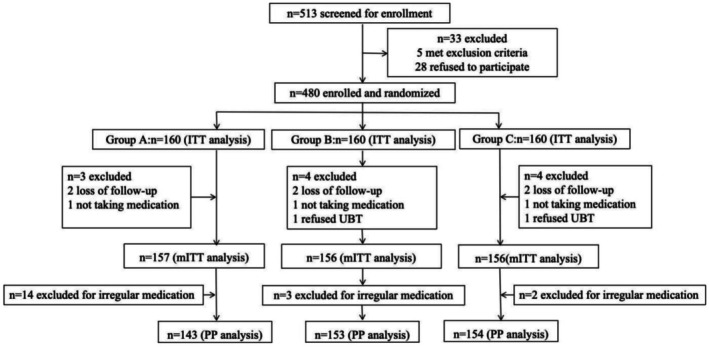
Study flowchart. *Note:* Group A, ilaprazole 5 mg quadruple group; Group B, ilaprazole 5 mg dual group; Group C, ilaprazole 10 mg dual group.ITT, intention‐to‐treat; mITT, modified ITT; PP, per‐protocol; UBT, urea breath test.

The study flowchart is presented in Figure 1. A total of 480 patients were included in this study, comprising 160 patients in each group. After excluding patients who were lost to follow‐up, did not undergo re‐examination after treatment, or failed to take the medication, 469 patients were included in the mITT analysis. In addition, after excluding noncompliant patients, a total of 450 patients were included in the PP analysis.

The baseline data before treatment are presented in Table 1. The differences in sex, age, smoking, alcohol intake, sharing tableware and sharing a cup among these groups showed no significant differences (all *p* > 0.05).

**TABLE 1:** Baseline characteristics of eligible individuals.

Baseline factors	Group A (*n* = 160)	Group B (*n* = 160)	Group C (*n* = 160)	Statistical value	p
Age [(mean, SD), y]	42.0 ± 12.0	41.5 ± 12.4	42.3 ± 11.4	0.143^a^	0.867
Sex (male/female)	83/77	73/87	88/72	2.917^b^	0.231
Smoking (yes/no)	30/130	25/135	23/137	1.194^b^	0.583
Alcohol intake (yes/no)	23/137	22/138	19/141	0.469^b^	0.837
Sharing tableware (yes/no)	148/12	146/14	149/11	0.410^b^	0.867
Sharing cup (yes/no)	12/148	7/153	13/147	2.076^b^	0.372

*Note:* Data are presented as mean ± SD or number; a is the F value; b is the χ^2^ value; Group A, ilaprazole 5 mg quadruple group; Group B, ilaprazole 5 mg dual group; Group C, ilaprazole 10 mg dual group.

Abbreviation: SD, standard deviation.

### 3.2 *H. pylori* Eradication Rates

**FIGURE 2:**
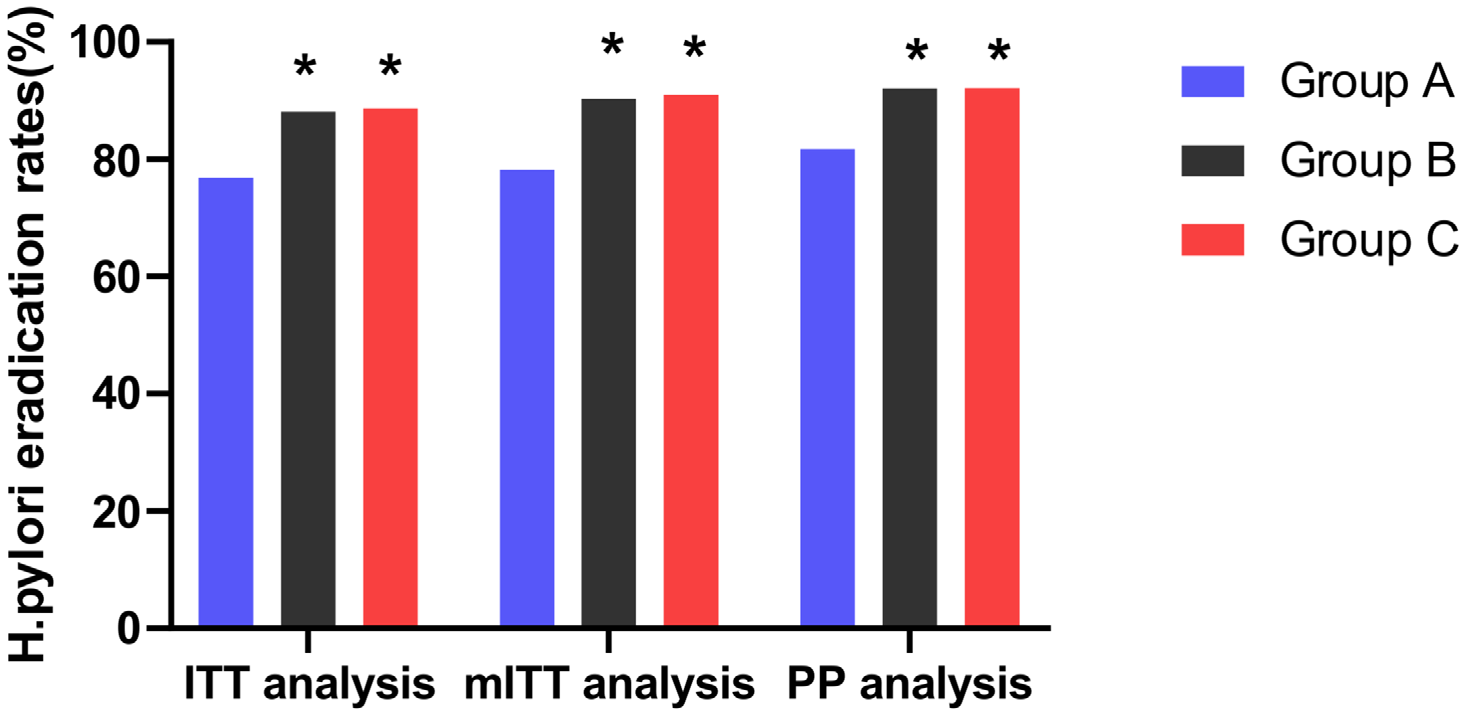
Comparison of *H. pylori* eradication rates. *Note:* *indicates that non‐inferiority was established compared with Group A; Group A, ilaprazole 5 mg quadruple group; Group B, ilaprazole 5 mg dual group; Group C, ilaprazole 10 mg dual group. ITT, intention‐to‐treat; mITT, modified ITT; PP, per‐protocol.

The eradication rates of *H. pylori* in the three treatment groups (A, B, and C) were analyzed using three different methods: ITT, mITT, and PP analyses. The results are summarized in Tables 2, 3 and Figure 2.

**TABLE 2:** Comparison of *H. pylori* eradication rates in each regimen.

Items	Group A	Group B	Group C	*χ* ^*2*^ value	*p*
ITT analysis	123/160 (76.9)	141/160 (88.1)	142/160 (88.7)	14.785	0.004
mITT analysis	123/157 (78.3)	141/156 (90.4)	142/156 (91.0)	13.752	0.001
PP analysis	117/143 (81.8)	141/153 (92.1)	142/154 (92.2)	10.611	0.005

*Note:* Data are presented as number (%); Group A, ilaprazole 5 mg quadruple group; Group B, ilaprazole 5 mg dual group; Group C, ilaprazole 10 mg dual group.

Abbreviations: ITT, intention‐to‐treat; mITT, modified ITT; PP, per‐protocol.

**TABLE 3:** Pairwise comparison of *H. pylori* eradication rates in each regimen.

Items	Group A	Group B	Group C
ITT analysis			
	123/160 (76.9)	141/160 (88.1)	142/160 (88.7)
Difference (95% CI) VS group A	—	11.2 (0.036, 0.193)	11.8 (0.037, 0.195)
Difference (95% CI) VS group B	—	—	0.6 (−0.067, 0.071)
mITT analysis			
	123/157 (78.3)	141/156 (90.4)	142/156 (91.0)
Difference (95% CI) VS group A	—	12.1 (0.043, 0.199)	12.7 (0.048, 0.208)
Difference (95% CI) VS group B	—	—	0.6 (−0.056, 0.064)
PP analysis			
	117/143 (81.8)	141/153 (92.1)	142/154 (92.2)
Difference (95% CI) VS group A	—	10.3 (0.022, 0.180)	10.4 (0.023, 0.186)
Difference (95% CI) VS group B	—	—	0.1 (−0.060, 0.066)

*Note:* Group A, ilaprazole 5 mg quadruple group; Group B, ilaprazole 5 mg dual group; Group C, ilaprazole 10 mg dual group.

Abbreviations: CI, confidence interval; ITT, intention‐to‐treat; mITT, modified ITT; PP, per‐protocol.

As shown in Table 2, the eradication rates in the ITT analysis were 76.9% (123/160) for group A, 88.1% (141/160) for Group B, and 88.7% (142/160) for Group C. In the mITT analysis, the eradication rates were 78.3% (123/157) for Group A, 90.4% (141/156) for Group B, and 91.0% (142/156) for Group C. In the PP analysis, the eradication rates were 81.8% (117/143) for group A, 92.1% (141/153) for Group B, and 92.2% (142/154) for Group C.

In the ITT dataset, the confidence intervals for the eradication rate differences between Group B and Group A, and between Group C and Group A, were (0.036, 0.193) and (0.037, 0.195), respectively. Non‐inferiority was established for both Group B and Group C (non‐inferiority margin: −0.1), with consistent conclusions observed across the ITT, mITT, and PP datasets. The specific data are shown in Table 3.

### 3.3 Adverse Events and Compliance

As listed in Table 4, the adverse event rates for patients in Group A, Group B and Group C were 28.0%, 10.2%, and 10.8%. The primary adverse events observed in the three groups were gastrointestinal symptoms, with the most common being a bitter taste. Medication compliance rates for patients in Group A, Group B, and Group C were 91.1%, 98.1%, and 98.7%, respectively.

**TABLE 4:** Comparison of adverse events and compliance among patients.

Safety and compliance	Group A (*n* = 157)	Group B (*n* = 157)	Group C (*n* = 157)	*χ* ^*2*^ value	*p*
Adverse events	44 (28.0)^†‡^	16 (10.2)^†§^	17 (10.8)^‡§^	23.505	< 0.001
Bitter taste	26 (16.6)^†‡^	2 (1.3)^†§^	2 (1.3)^‡§^	41.012	< 0.001
Diarrhea	4 (2.5)	3 (1.9)	4 (2.5)	0.291	0.868
Bloating	4 (2.5)	4 (2.5)	3 (1.9)	0.291	0.868
Anorexia	3 (1.9)	2 (1.3)	3 (1.9)	0.390	0.828
Rash	2 (1.3)	1 (0.6)	1 (0.6)	0.673	0.702
Nausea	5 (3.2)	4 (2.5)	4 (2.5)	0.244	0.885
Compliance	143 (91.1)^¶+^	154 (98.1)^§+^	155 (98.7)^¶§^	14.588	0.001

*Note:* Data are presented as number (%); ^†^A vs. B, *P* < 0.001; ^‡^A vs. C, *P* < 0.001; ^§^B vs. C, *P* > 0.05; ^¶^A vs. C, *p* < 0.01; ^+^A vs. B, *p* < 0.05; Group A, ilaprazole 5 mg quadruple group; Group B, ilaprazole 5 mg dual group; Group C, ilaprazole 10 mg dual group.

### 3.4 Impact of Baseline Data on the Eradication Rate

As shown in Table 5, sex, smoking, alcohol intake, sharing tableware, and sharing cups of the patients had no effect on the effectiveness of the three regimens. Compliance is positively associated with *H. pylori* eradication rates.

**TABLE 5:** Factors influencing eradication rates in the mITT population.

Influencing factor	Eradication situation		
	Group A (*n* = 157)
Group B (*n* = 156)
Group C (*n* = 156)
Sex			
Male	63/82	65/71	77/86
Female	60/75	76/85	65/70
OR (95% CI)	0.889 (0.488–1.618)	1.126 (0.497–2.551)	0.591 (0.238–1.468)
Smoking			
Yes	22/29	23/25	18/23
No	101/128	118/131	124/133
OR (95% CI)	0.801 (0.379–1.694)	2.644 (0.588–11.894)	0.304 (0.092–1.000)
Alcohol intake			
Yes	15/23	20/21	16/19
No	108/134	121/135	126/137
OR (95% CI)	0.550 (0.250–1.212)	2.131 (0.469–9.685)	0.465 (0.145–1.488)
Sharing tableware			
Yes	112/145	129/142	132/145
No	11/12	12/14	10/11
OR (95% CI)	0.321 (0.069–1.494)	1.806 (0.537–6.070)	1.108 (0.131–9.409)
Sharing Cup			
Yes	10/12	6/7	10/12
No	113/145	135/149	132/144
OR (95% CI)	1.360 (0.409–4.515)	0.574 (0.110–2.999)	0.414 (0.080–2.128)
Compliance			
Good	117/143	140/153	141/154
Poor	6/14	1/3	1/2
OR (95% CI)	4.370 (1.558–12.260)	23.500 (3.897–141.711)	11.833 (1.529–91.610)

*Note:* Data are presented as numbers; Group A, ilaprazole 5 mg quadruple group; Group B, ilaprazole 5 mg dual group; Group C, ilaprazole 10 mg dual group.

Abbreviations: CI, confidence interval; OR, odds ratio.

## 4 Discussion

In China, the application of bismuth‐containing quadruple therapy faces numerous issues, mainly related to the decreased eradication rate caused by antibiotic resistance, drug side effects, and patient compliance [12]. The reported antibiotic resistance rates for clarithromycin, levofloxacin, and metronidazole are high in China, ranging from 20% to 40%. Specifically, the resistance rate for metronidazole can reach 60%–90%, whereas amoxicillin has a low resistance rate, ranging from 0% to 7% [13].

High‐dose amoxicillin dual therapy represents a novel and promising alternative that has been extensively researched in recent years [14, 15]. Research indicates that increasing the dosage of PPIs leads to improved eradication rates of *H. pylori* . This improvement is likely due to the impact that amoxicillin's eradication efficacy heavily depends on the pH level of the gastric environment. An in vitro study revealed that the MIC_90_ of the *β*‐lactam antibiotic ampicillin significantly increases as pH levels rise: it is 2 μg/mL at a pH range of 5.7 to 6.0, 0.5 μg/mL at pH 7.4, and 0.25 μg/mL at pH levels between 7.8 and 8.0. Research has indicated that as the intragastric pH increases, both the stability and bacteriostatic effect of amoxicillin are enhanced. Furuta T et al. [16] conducted a comparison of eradication rates for various dosages of rabeprazole in dual therapy regimens. They discovered that administering rabeprazole at 20 mg three times daily resulted in a higher eradication rate than a regimen of 10 mg twice daily, with the same dosage of amoxicillin (ITT: 89.8% versus 75.9%; PP: 93% versus 80%). Therefore, the selection of PPIs with strong and long‐lasting acid inhibitory effects is essential to improve the eradication rate of *H. pylori* .

Ilaprazole, a third‐generation PPI, is not metabolized by the CYP2C19 enzyme, which results in reduced individual variability and long‐lasting acid inhibition. Shin JS et al. [17] found that ilaprazole provided better 24 h pH control than esomeprazole in healthy subjects, especially during the evening and overnight periods. A meta‐analysis [18] published in 2024 has indicated that ilaprazole provides better performance in terms of nocturnal acid inhibitory effects compared to other PPIs. In China, the “Fifth National Consensus Report on the Management of *Helicobacter pylori* Infection” published in 2017, recommended the ilaprazole use of 5 mg twice a day as a viable option within the bismuth‐based quadruple therapy regimen [19].

Recent studies on ilaprazole in dual therapy for *H. pylori* eradication have shown promising results. Feihu Bai et al. [20] found that compared to the bismuth‐based quadruple therapy with ilaprazole (IAFB), the ilaprazole dual therapy (IA, Ilaprazole: 5 mg bid) was equally effective, safer, and more cost‐effective (ITT: 92.1% vs. 91.9%, *p* = 1; PP: 94.9% vs. 93.6%, *p* = 0.765). This conclusion was also confirmed in another study by Feihu Bai et al. [8] (IAFB‐14d, IAFB‐10d, and IA‐14d groups: ITT: 84.0%, 79.3%, and 88.0%, PP: 94.7%, 87.5%, and 93.0%). Jianping Cheng et al. [21] compared high‐dose ilaprazole (10 mg bid) dual therapy with standard ilaprazole bismuth‐based quadruple therapy, finding that 14‐day ilaprazole high‐dose therapy was more effective in *H. pylori* eradication, with improved safety and compliance (ITT/PP: 76.3% vs. 61.3%, *p* = 0.015). However, the overall eradication rate in this study was relatively low, which may be attributed to the centralized procurement of amoxicillin [22] and the high rate of clarithromycin resistance in the Beijing area [23]. For this study, the selected amoxicillin has undergone a pharmacokinetic consistency evaluation, confirming its high quality. The outcome also showed its good performance in clinical practice. Further research is needed to confirm these findings. However, Kwack et al. [24] reported unsatisfactory eradication with high‐dose dual therapy (ilaprazole 40 mg twice daily plus amoxicillin 750 mg four times daily; 79.3% ITT, 82.1% PP). In contrast, our trial achieved higher eradication with much lower ilaprazole doses (5–10 mg twice daily). A key difference between that trial and our study is the sample size and study design. Although the nominal ilaprazole dose was higher in the Korean study, the small sample size and single‐center design likely limited the generalizability and statistical reliability of the findings. In addition, the difference may also be attributed to optimized acid suppression and amoxicillin exposure rather than nominal ilaprazole dose. Higher ilaprazole may not proportionally increase acid suppression beyond a ceiling effect. Instead, it may increase drug cost and adverse events without efficacy benefit. Additional factors include regional resistance; importantly, our multicenter design with adherence monitoring and standardized administration. Collectively, these findings highlight that pharmacodynamic optimization and protocol fidelity are more critical to dual therapy success than dose escalation.

Our study was the first exploration of various doses of ilaprazole for *H. pylori* eradication. It was discovered that the eradication rates for both the standard and double‐dose groups of ilaprazole dual therapy were statistically superior to those of the bismuth‐containing quadruple therapy. In line with other reported studies, the incidence of adverse drug events associated with ilaprazole dual therapy is statistically significantly lower in both the standard and double‐dose groups (10.2% and 10.8%, respectively) than in the bismuth‐containing quadruple therapy group (28.0%, *p* < 0.001). Compliance was also statistically significantly higher in either dual therapy group (98.1% and 98.7%, respectively) than in the bismuth‐containing quadruple therapy group (91.1%, *p* = 0.001). To our surprise, no statistically significant differences were observed between the two dosage groups of ilaprazole dual therapy (*p* > 0.05) in terms of efficacy, safety, or compliance. Although stronger acid suppression is generally expected to improve eradication rates, in our study the 10 mg twice‐daily ilaprazole regimen did not achieve better outcomes than the 5 mg twice‐daily regimen. Considering the consistently low and stable resistance of *H. pylori* to amoxicillin in the Beijing area, these results indicate that the standard 5 mg twice‐daily dose of ilaprazole is sufficient to maintain intragastric pH at levels optimal for amoxicillin activity. Therefore, standard doses of ilaprazole may fulfill the therapeutic requirements adequately.

In our study, dual therapy demonstrated a greater potential for clinical application. However, considering that all participating centers were situated in Beijing, these findings are solely applicable to the conditions in that city. The improved eradication rate of dual therapy can likely be attributed to the low resistance to amoxicillin and the high resistance to clarithromycin, especially in Beijing. Antibiotic resistance exhibits distinct regional differences in China. Chen et al. [4] analyzed previous data up to the year 2020 and found that the clarithromycin resistance in northern (37%, 95% CI: 32%–41%) and western China (34%, 95% CI: 17%–54%) was higher than that in eastern (24%, 95% CI: 20%–28%) and southern China (24%, 95% CI: 17%–32%) (*p* = 0.0004). Over the past 20 years, a significant upward trend in clarithromycin resistance has been observed in both the northern and eastern regions, with an increase from 16% to 42% (*p* < 0.0001) in the north and from 20% to 30% (*p* = 0.008) in the east. Li et al. conducted a review indicating that the prevalence of amoxicillin resistance has remained relatively low, not exceeding 1.2% (*p* = 0.631), across various regions and has been stable over the past twenty years. When comparing the primary and secondary resistance rates for the antibiotics under discussion, a sharp increase in drug resistance was observed, rising from 29% to 46% between 2016 and 2020 (*p* = 0.001) for secondary clarithromycin treatment in the Beijing area. However, the secondary resistance rate for amoxicillin remained low, with no significant increase (*p* = 0.094) compared to its primary resistance rate. With respect to the history of previous medication, Li et al. noted that the resistance rates for clarithromycin increased sharply (*p* < 0.001) to high levels upon repeated use (from 41.4% to 80.0%), whereas amoxicillin maintained a low resistance rate upon repeated medication (from 0.7% to 9.5%, *p* < 0.001) in the Beijing area [23].

In the mITT population, subgroup analyses were performed to explore the potential influence of demographic and behavioral factors, including sex, smoking, alcohol intake, and the habit of sharing tableware or cups, as well as medication adherence, on eradication outcomes across the three regimens (Table 5). None of the demographic or behavioral variables were significantly associated with treatment success. Conversely, medication adherence exhibited a strong positive association with eradication efficacy. This finding underscores that, irrespective of the specific dosing regimen, strict adherence to therapy remains a pivotal determinant of successful *H. pylori* eradication.

There were still some limitations to this study. First, it only involved initial eradication patients for *H. pylori* infection; the results of standard doses for retreatment patients remain uncertain. Secondly, the participants in this study were recruited from the Beijing region, where amoxicillin demonstrated a low resistance rate. Further research is warranted to ascertain the generalizability of these findings to more extensive populations and geographic areas, as well as to evaluate the necessity for higher ilaprazole dosages across a broader range of conditions. Thirdly, this study did not assess *H. pylori* antibiotic resistance, particularly to clarithromycin, which may explain the suboptimal efficacy observed in the quadruple therapy group. Moreover, previous exposure to clarithromycin was not considered during treatment group assignment, potentially influencing eradication outcomes. Future studies should integrate resistance profiling to guide personalized regimens and, in settings where susceptibility testing is unavailable or infeasible, clinicians should carefully consider patients' prior exposure to antimicrobials when selecting first‐line treatment strategies. Additionally, the study did not include an analysis of cost‐effectiveness, which is an important factor in clinical decision‐making. The limited global availability of ilaprazole also restricts the applicability of these findings to regions where this drug is accessible.

In summary, administering standard or double doses of ilaprazole twice daily for dual therapy has demonstrated superior efficacy, safety, and patient compliance compared to the traditional bismuth‐containing quadruple therapy regimen. It is anticipated to become a potential first‐line treatment for *H. pylori* infection.

## Author Contributions

Conception and design of the research: Y.C., S.D.i., Q.H., M.Z., S.D.u., Y.G., Y.Z. Acquisition of data: Q.H., M.Z., S.D.u., Y.G., Y.Z., Y.W., X.L., H.G. Analysis and interpretation of the data: Q.H., M.Z. Statistical analysis: Q.H., M.Z. Obtaining financing: Y.C., S.D.i. Writing of the manuscript: Q.H., M.Z. Critical revision of the manuscript for intellectual content: Y.C., S.D.i. All authors read and approved the final draft.

## Conflicts of Interest

The authors declare no conflicts of interest.

## Data Availability

The data that support the findings of this study are available from the corresponding author upon reasonable request.
